# After the virus has cleared—Can preclinical models be employed for Long COVID research?

**DOI:** 10.1371/journal.ppat.1010741

**Published:** 2022-09-07

**Authors:** Ethan B. Jansen, Spencer N. Orvold, Cynthia L. Swan, Anthony Yourkowski, Brittany M. Thivierge, Magen E. Francis, Anni Ge, Melissa Rioux, Joseph Darbellay, John G. Howland, Alyson A. Kelvin

**Affiliations:** 1 Vaccine and Infectious Disease Organization VIDO, University of Saskatchewan, Saskatoon, Saskatchewan, Canada; 2 Department of Biochemistry, Microbiology, and Immunology, University of Saskatchewan, Saskatoon, Saskatchewan, Canada; 3 Department of Anatomy, Physiology, and Pharmacology, University of Saskatchewan, Saskatoon, Saskatchewan, Canada; 4 Department of Microbiology and Immunology, Faculty of Medicine, Dalhousie University, Halifax, Nova Scotia, Canada; University of Pittsburgh, UNITED STATES

## Abstract

Severe Acute Respiratory Syndrome Coronavirus (SARS-CoV-2) can cause the life-threatening acute respiratory disease called COVID-19 (Coronavirus Disease 2019) as well as debilitating multiorgan dysfunction that persists after the initial viral phase has resolved. Long COVID or Post-Acute Sequelae of COVID-19 (PASC) is manifested by a variety of symptoms, including fatigue, dyspnea, arthralgia, myalgia, heart palpitations, and memory issues sometimes affecting between 30% and 75% of recovering COVID-19 patients. However, little is known about the mechanisms causing Long COVID and there are no widely accepted treatments or therapeutics. After introducing the clinical aspects of acute COVID-19 and Long COVID in humans, we summarize the work in animals (mice, Syrian hamsters, ferrets, and nonhuman primates (NHPs)) to model human COVID-19. The virology, pathology, immune responses, and multiorgan involvement are explored. Additionally, any studies investigating time points longer than 14 days post infection (pi) are highlighted for insight into possible long-term disease characteristics. Finally, we discuss how the models can be leveraged for treatment evaluation, including pharmacological agents that are currently in human clinical trials for treating Long COVID. The establishment of a recognized Long COVID preclinical model representing the human condition would allow the identification of mechanisms causing disease as well as serve as a vehicle for evaluating potential therapeutics.

## Author summary

Not everyone who develops COVID-19 (Coronavirus Disease 2019) recovers completely after 5 days, 14 days, or even 12 weeks. People who experience continuing or new symptoms such as fatigue, difficulty breathing, joint pain, heart dysfunction, and memory issues are now recognized as having “Long COVID” or Post-Acute Sequelae of COVID-19 (PASC). Although Long COVID has been estimated to affect 30% to 75% of recovering COVID-19 patients in some studies, the illness is poorly understood, leaving us unable to properly support patients in their recovery. Here, we summarize COVID-19 and Long COVID in people then discuss the potential utility of Severe Acute Respiratory Syndrome Coronavirus (SARS-CoV-2) animal models to address problems of Long COVID. We review SARS-CoV-2 infection and disease manifestations in mice, Syrian hamsters, ferrets, and nonhuman primates (NHPs). Specifically, the virology, pathology, immune responses, and multiorgan involvement are summarized. These models are rationally examined for their use in understanding the complexities of the multiorgan involvement during Long COVID and the potential for their use in screening therapeutics such as anti-inflammatory drugs, which are currently used for treating other diseases. The aim of this review is to highlight Long COVID preclinical animal models that could be used for Long COVID research and to screen for pharmacological interventions.

## Introduction

Not everyone who develops COVID-19 (Coronavirus Disease 2019) recovers completely after 5 days, 14 days, or even 12 weeks. The SARS-CoV-2 (Severe Acute Respiratory Syndrome Coronavirus 2) virus, which causes COVID-19, initially infects epithelial cells of the respiratory tract and typically resolves within 2 weeks of infection [1–3]. However, after the virus has been cleared in the acute phase, people can continue experiencing new or prolonged symptoms such as fatigue, dyspnea, arthralgia, cardiac dysfunction, and memory issues [4–7]. People experiencing prolonged symptoms are now recognized as having Long COVID or Post-Acute Sequelae of COVID-19 (PASC) [7–9]. Strikingly, some studies have indicated that between 30% to 75% of recovering COVID-19 patients may experience Long COVID symptoms [6,10]. Critically, as Long COVID is a newly recognized disease, the clinical picture and specific diagnostic criteria are not well defined. Furthermore, understanding of the molecular mechanisms leading to disease development is also poor. Preclinical models are the cornerstone of identifying disease mechanisms under controlled experimental conditions, especially for emerging virus research. The use of preclinical models not only enables the study of disease mechanisms in an intact system of the organism but also guides the discovery and evaluation of correlates of disease and therapeutic targets. As the number of people who become infected with SARS-CoV-2 and recover increases, it is essential to recognize that healthcare systems now must respond to a possibly large number of people suffering from prolonged disease. To enhance the response to current and future waves of Long COVID patients, we feel it is essential to have preclinical models that recapitulate the symptoms and disease pathogenesis of Long COVID in humans.

Here, we review SARS-CoV-2 infection in Syrian hamster, mouse, ferret, and nonhuman primates (NHPs), and the associated clinical disease as well as immune responses. These models are rationally examined for their use in understanding the complexities of multiorgan involvement during Long COVID as well as the potential for specific pharmacological treatments to create a framework for a Long COVID preclinical model.

### Severe Acute Respiratory Syndrome Coronavirus 2 (SARS-CoV-2) and acute COVID-19

SARS-CoV-2, the cause of COVID-19 which led to the COVID-19 pandemic, is a lipid enveloped, single-stranded positive-sense RNA *Betacoronavirus* of the family Coronaviridae [11]. The viral genome of SARS-CoV-2 encodes 12 functional open reading frames. As a respiratory virus, SARS-CoV-2 initially targets epithelial cells such as type pneumocytes of the respiratory tract with common symptoms such as cough, fever, fatigue, dyspnea, upper respiratory complications [12,13], and less common symptoms such as loss of smell, neurological sequelae, and micro blood clots [14–18]. COVID-19 varies in clinical severity in humans, ranging from asymptomatic, mild, or moderate to severe disease, and death [1]. Host characteristics and comorbidities have been linked to increased severity risk as well as dysregulated immune responses [19,20]. Host responses and immune activation play a crucial role in determining the outcome of SARS-CoV-2 infection and COVID-19 severity. Like other coronaviruses, SARS-CoV-2 has mechanisms to evade type I and III interferons (IFNs) [21] and COVID-19 patients with severe disease have suppressed or delayed IFN responses [22,23]. Delayed type I IFN response allows the virus to replicate to higher levels and initiate significantly strong innate and inflammatory immune responses, leading to inflammatory cell migration in the lung and cytokine storm [24]. Studies of the adaptive immune response to SARS-CoV-2 indicate classical B cell and T cell initiation regulating virus clearance and establishing pathogen-specific immune memory [24–27]. Studies on immune durability indicate that SARS-CoV-2-specific antibodies are relatively stable over 5 months post-symptom onset (pso), while the compartments of the memory response (B cells, CD8+ T cells, CD4+ T cells) fluctuated over time [28,29]. However, it is still unclear how long individuals sustain neutralizing antibodies and effective memory cell repertoires. More studies are needed to determine if immune regulation plays a role in the sequelae of Long COVID.

### Long COVID or Post-acute Sequelae of COVID-19 (PASC)

**Fig 1:**
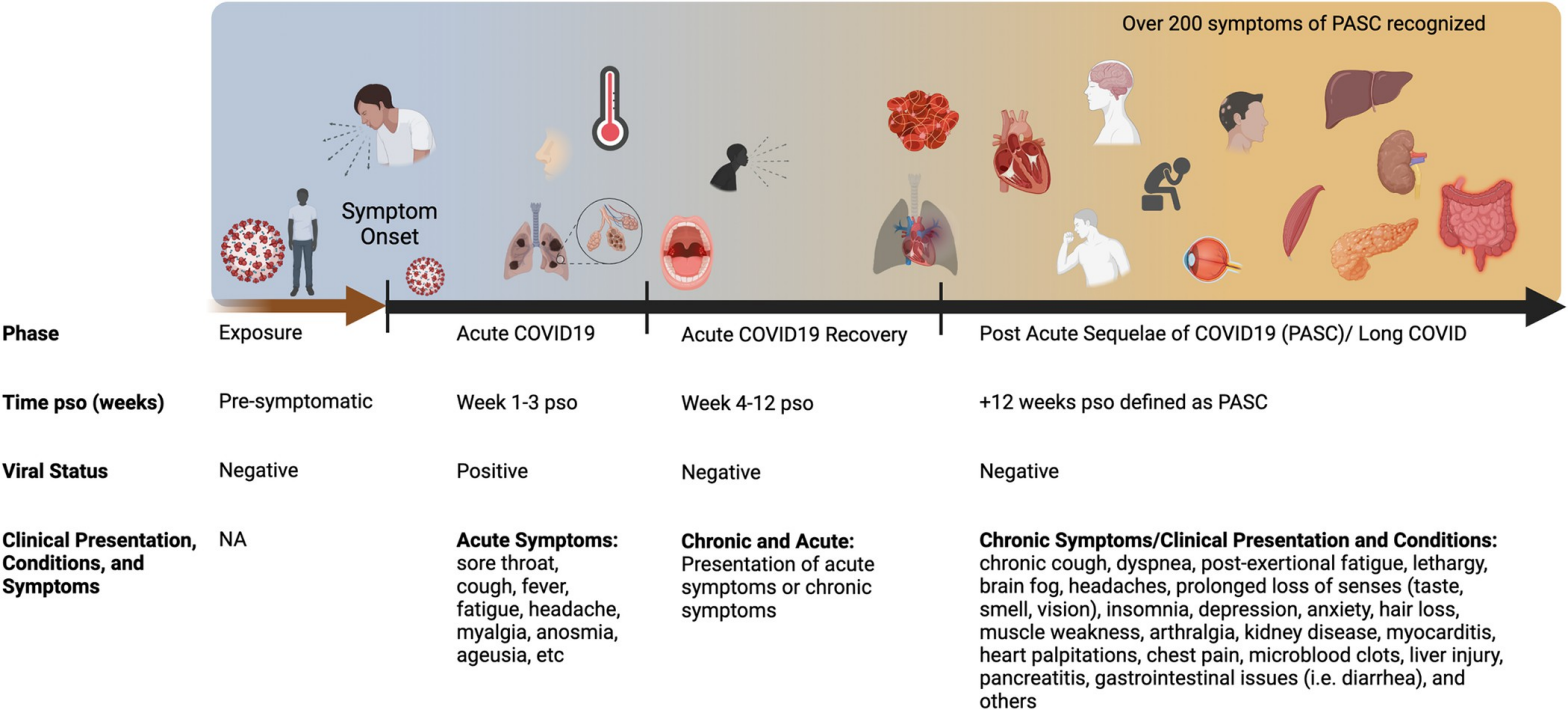
Time course and symptoms of COVID-19 and PASC. Acute COVID-19 includes symptoms present up to 4 weeks. Live virus is typically present in the respiratory tract for 1-week pso, with variability among individuals. Common symptoms of acute COVID-19 are summarized that include pulmonary and systemic manifestations. Long COVID or PASC includes persistent symptoms present 4 weeks post-acute infection. The diverse range of multiorgan complications of PASC are summarized. COVID-19, Coronavirus Disease 2019; PASC, Post-Acute Sequelae of COVID-19; pso, post-symptom onset.

The long-term effects of COVID-19 are becoming more apparent as patients continue to report lingering symptoms, known under various names including Long COVID, post-acute COVID-19, or PASC [30]. Some PASC symptoms overlap with symptoms reported by patients experiencing chronic viral fatigue (CVF) after SARS-CoV or Middle East Respiratory Syndrome (MERS) infection [5,31]. Although PASC patients report similar fatigue-related symptoms to CVF, there is a different range of symptoms involving multiple organs indicating that PASC is not synonymous with CVF [31]. Since PASC is newly identified and not well understood, the condition does not have an internationally accepted definition. The National Institute for Health and Care Excellence (NICE) in the United Kingdom references 2 stages of PASC; 4 to 12 weeks pso and 12+ weeks pso [7]. The second stage of PASC, where symptoms continue to be experienced after 12 weeks is in line with the World Health Organization’s (WHO) definition [32]. For this review, we will include complications 4 weeks pso in our discussion to encompass both stages (Fig 1).

PASC is a multiorgan disease that can cause complications in the lungs, heart, brain, spleen, liver, blood vessels, gastrointestinal tract, kidney, and pancreas as well as other organs [33]. Common symptoms include fatigue, headaches, cognitive disturbances, dyspnea, cough, heart palpitations, chest pains, myocarditis, and muscle pain (summarized in Fig 1) [33–35]. In addition, there are other less common sequelae, including pancreatitis, nausea, diarrhea, sore throat, kidney injury, depression, anxiety, splenic complications, cardiovascular abnormalities, and olfactory dysfunction [33]. Multisystem inflammatory syndrome in children (MIS-C) is also reported after acute COVID-19 in some cases; however, MIS-C is typically not considered to be PASC or associated with Long COVID [36–38].

Although PASC is now recognized as a condition following acute COVID-19, the mechanisms leading to long-term organ-specific damage due to SARS-CoV-2 infection and the development of PASC in humans are still relatively unknown. There are 3 main mechanisms hypothesized to drive PASC: (1) viral persistence; (2) prolonged inflammation; and (3) viral-induced autoimmunity [4,8]. Additional pathophysiological factors have also been suggested as the cause of the disease but are less prominently considered. These factors include immune exhaustion, metabolic imbalances, microblood clots, and an altered microbiome [4,8]. A study following patients with Long COVID found highly activated innate immune cells and elevated expression of proinflammatory cytokines in serum up to 8 months pi suggesting a role for dysregulated inflammation [39]. Furthermore, a recent study indicated the persistence of the SARS-CoV-2 S1 protein in nonclassical proinflammatory monocytes [40]. To explore these clinical findings more in-depth, animal models will be essential for elucidating the systemic and organ-specific mechanisms of post-acute COVID-19.

Prevalence estimations of post-acute COVID-19 vary and are hampered by differences in the survey methods employed in each study. A Canadian study using patient-reported outcome measures (PROMs) to assess the quality of life of hospitalized patients found that over 50% had reduced quality of life 3 months after acute infection [41]; however, the study only captured the effects on patients with more severe acute disease (hospitalized patients). A more extensive study from the UK Office of National Statistics surveyed over 8,000 respondents who tested positive for COVID-19 but were not hospitalized [42]. The authors found that 21% of respondents were symptomatic at week 5 pi, and 9.9% were symptomatic at 12 weeks pi [42]. Although the prevalence of Long COVID varied in these studies, we can conclude that a portion of COVID-19 survivors will need ongoing care for a broad range of persistent symptoms. Also, the number of people needing long-term support will grow as people continue to be infected with the SARS-COV-2 virus highlighting the necessity of Long COVID research.

Although susceptibility to PASC is not entirely clear, some studies suggest it may be more common in people with severe acute disease, such as those who have been hospitalized [43]. A correlation between ICU patients or patients who required ventilation with the development of PASC has been observed [5,44,45]. However, other reports found that patients who exhibit mild or moderate acute phase symptoms can also potentially suffer long-term symptoms [46]. One study identified that persistent COVID-19 symptoms may be more greatly associated with the experience of more than 5 acute phase symptoms, not necessarily the severity [47]. The 5 key symptoms included hoarseness of voice, fatigue, headache, dyspnea, and myalgia [47].

### Host factors and biomarkers of PASC susceptibility

Host factors and specific vulnerable populations have been associated with the development of PASC. For example, age and sex, as well as chronic conditions or comorbidities such as diabetes, hyperglycemia, obesity, cardiovascular disease, asthma, and renal disease are also linked to PASC [6,47–49]. One hypothesis suggests that the chronic state of inflammation associated with these diseases may initiate excessive inflammatory cytokine levels during acute SARS-CoV-2 infection that can lead to long-term damage of internal organs and prolonged symptoms [48,50]. In the case of obesity, excessive inflammation is caused by adipocyte production of inflammatory adipokines (fat tissue-associated cytokines) such as interleukin (IL)-8, plasminogen activator inhibitor (PAI)-1, monocyte chemoattractant protein (MCP)-1, IL-6, tumor necrosis factor-alpha (TNF-α), and IL-18. Additionally, the adipokine serum amyloid-A can amplify macrophage production of TNF-α, IL-1, and IL-6, contributing to the chronic inflammatory state [50].

Biomarkers in Long COVID patients have also been investigated as possible predictors for developing PASC [30]. A study in California suggested that patients who went on to develop PASC had a higher level of the proinflammatory cytokines TNF-α and IP-10 during early recovery from acute COVID-19 [51]. Other biomarkers such as elevated blood urea, high D-dimer levels, C reactive proteins, and low lymphocyte levels have also been linked to long-term pulmonary, heart, and liver abnormalities several months after acute COVID-19 [30]. However, investigations on biomarkers and immune mediators in Long COVID are not consistent, and this remains an active area of interest [44,52].

### Animal models of SARS-CoV-2

The development of suitable animal models for PASC will be essential for addressing the complex mechanisms of multiorgan sequelae as well as developing therapeutics. Some preclinical models may be better than others for specific tasks such as determining the roles of age, sex, and specific organ involvement in the development and severity of Long COVID. Below, we review the animal models found to be susceptible to SARS-CoV-2 infection for their potential use in PASC research.

### Syrian hamster model

Syrian hamsters (*Mesocricetus auratus*) have emerged as a useful model of SARS-CoV-2 for testing vaccines [53–56] and antivirals [57–60], as well as determining mechanisms of acute disease progression [61–70]. As Syrian hamsters have previously been used in the study of SARS-CoV [64,71] due to the similar expression profile of ACE2 between hamsters and humans, they were among the first animals investigated as a COVID-19 model [72]. SARS-CoV-2 infection in Syrian hamsters led to nonlethal disease and the clinical presentation of rapid weight loss and severe lung pathology were similar to those symptoms seen in acute COVID-19 in humans (Table 1) [73]. The viral dynamics, pathology, and immune responses within the respiratory tract have been well investigated [74–76]. Multiple studies indicate that live viral infection in the lung peaks early in the infectious time course (approximately day 2 pi) in hamsters prior to resolution approximately day 7 pi [62,63,67,69,70,73].

**Table 1:** Overview of commonly utilized SARS-CoV-2 animal models.

Animal	Disease severity	Clinical signs	Virus replication	Tissue pathology	Mortality	Ref.
Syrian Hamsters	Mild to moderate	Weight loss, decrease in temperature early	**Live virus:** Upper respiratory tract, trachea, lungs, mediastinal lymph node **Viral RNA:** Upper respiratory tract, trachea, lungs, mediastinal lymph node, heart, kidney, liver, spleen, large intestine, brain	Lung damage: hemorrhage and mononuclear cell infiltration in alveolar space, hyaline membrane formation, type II pneumocyte hyperplasia Myocarditis, microthrombi in the heart Tubular inflammation in the kidney	Low	[62,67,70,73,77]
Mice K18-hACE2	Mild to severe	Weight loss, lethargy, hunched posture, labored breathing, ruffled fur	**Live virus:** Upper respiratory tract, lungs, brain, small intestine, and large intestine **Viral RNA:** Upper respiratory tract, lungs, heart, brain, kidney, spleen, intestines	Lung damage: extensive inflammation, abundant immune cells in the alveolar, and interstitial locations of lung parenchyma, edema, fibrin deposition, and lung consolidation Cell death and thrombi in the thalamus, encephalitis, extensive N protein staining in brain regions with cell death and debris	Variable; dose-dependent	[78–82]
hACE2 Knock-in (KI)	Mild	No significant weight loss	**Live virus:** Nasal turbinate, lungs **Viral RNA:** Nasal turbinate, lungs, olfactory bulb, and brain in some animals	Limited lung inflammation, increase of immune cells in alveolar spaces	None reported	[80,83]
Ad5-hACE2	Mild	Minimal clinical signs of infection, some reports of weight loss with higher doses	**Live virus:** Nasal turbinate, lungs, **Viral RNA:** Lungs, heart, spleen, and brain	Lung inflammation (lymphoplasmacytic with few neutrophils)	None reported	[81,84]
Ferrets	Mild	Elevated body temperature lethargy, nasal discharge, sneezing	**Live virus:** Nasal turbinate, trachea, lungs (low) **Viral RNA:** Nasal turbinate, tonsil, trachea, lungs (low), intestine, kidney, feces, urine	Mild necrosis of epithelial cells in the nasal cavity Mild bronchiolitis and viral pneumonia (high dose)	None reported	[22,85–87]
NHPs	Mild to moderate	Weight loss, reduced appetite, lethargy, changes in respiratory pattern, elevated temperature early	**Live virus:** Nasal swab, throat, lungs **Viral RNA:** Nasal wash, tonsil, trachea, lungs, ileum, colon, kidney, liver, spleen, mesenteric lymph node, testicle, brain, spinal cord, rectal swab	Mild to moderate interstitial pneumonia, alveolar necrosis, alveolar edema, type II pneumocyte hyperplasia, red patchy lesions, ground glass opacity Multifocal and perivascular foci of moderate lymphocytic infiltrates in the conjunctiva Lymphoid hyperplasia	None reported	[88–93]

NHP, nonhuman primates; SARS-CoV-2, Severe Acute Respiratory Syndrome Coronavirus.

Histological and CT examination of the lungs in response to SARS-CoV-2 infection revealed a consistent damage and recovery profile in Syrian hamsters. Severe lung abnormalities were commonly first detected on day 2 pi [63,70]. Multiple studies observed mononuclear cell infiltration in areas where viral antigen was detected [63,67,70]. Viral protein was abundantly expressed in bronchiolar epithelial cells [63,70], alveolar macrophages, and type I and II pneumocytes [62]. Focal inflammatory cell infiltration in the interstitium and the alveolar cavity were most severe on days 7 or 8 pi, and pulmonary edema and alveolar hemorrhage were evident in some areas of the lungs [62,63,70]. Viral-induced damage in the lung on day 2 pi was ill-defined with patchy ground-glass opacity (GGO) central and peribronchially distributed [63]. Lung abnormalities progressed to more severe with rounded, multilocular GGO and regions of lung consolidation by days 7 to 8 pi [63]. Another study described infected animals as exhibiting typical histopathological signs of necro-suppurative pneumonia with suppurative bronchitis, necrosis of bronchial epithelial cells, and endothelialitis on day 3 pi that progressed to bronchial hyperplasia, severe interstitial pneumonia with marked type II alveolar epithelial cell hyperplasia, and endothelialitis by day 5. In all studies, acute damage was mostly resolved by day 14 pi, although chronic features of repair and long-term pathology such as hyaline membrane formation [70], alveolar cell hyperplasia, bronchiolization, increased fibroblasts, and presence of macrophage aggregates were still present [69].

The lung damage identified by histopathology is accompanied by specific immune gene regulation [63,70]. IFN-β was significantly down-regulated in both the nasal turbinate and right cranial lung, with the most significant inhibition on day 2 in SARS-CoV-2 infected Syrian hamsters [70]. Conversely, IFN-γ was up-regulated in the lung, resolving by day 15 pi [70]. Similar trends were also observed for inflammatory genes such as TNF-α [63]; however, this varied by hamster age [94–96]. A marked increase in CD3 has also been noted in multiple studies on days 5/6 pi [67,96]. CXCL10 has been reported to increase in the acute period but not return to baseline levels by day 15 pi [96]. Together, these studies suggest hamsters have signs of recovery from SARS-CoV-2 infection by day 15, although pathological disease signatures still persist in the lung.

Cardiovascular-related outcomes of COVID-19 have also been explored in hamsters. Live virus has not been detected in the heart (heavily in cardiomyocytes) after infection; however, the presence of viral RNA has been detected and found to persist throughout the acute time course [62,69,70,97]. Our group found inflammatory cytokine gene expression, such as TNF-α and IL-6, to be increased in the heart. We also identified IL-4 and IL-5 to be increased in the heart which we associated with eosinophilic myocarditis noted in the histopathological analysis. Additionally, IL-2 and IL-10 were increased possibly suggesting monocyte-induced damage [70,98,99]. Histopathological analysis of the heart also revealed microthrombi and myocarditis in our study [70]. Other groups have found mild focal myocardial degeneration and interstitial edema [62], and a recent study demonstrated cardiovascular complications (CVCs) in hamsters, ventricular hypertrophy and thickening, coronary fibrosis, and increased inflammation in the heart on days 7 and 14 pi [100]. In summary, SARS-CoV-2 studies in hamsters consistently demonstrate damage to the heart.

Central nervous system dysfunction has also been described in the Syrian hamster model in response to SARS-CoV-2; however, whether these effects are direct causes of infection in the central nervous system is contentious [101,102]. Several studies have indicated that ACE2 in hamsters is present in the olfactory epithelium and sustentacular cells that support the olfactory neurons, rather than in the olfactory neurons themselves [103,104]. This observation suggests indirect regulation of the central nervous system. An additional study has also found SARS-CoV-2 RNA in the brain, although the IHC staining for the nucleocapsid protein was negative indicating the absence of replicating virus [63]. However, another report indicated that SARS-CoV-2 could directly infect Syrian hamster olfactory sensory neurons [67]. Although there have been conflicting thoughts surrounding SARS-CoV-2 direct infection in the brain, viral RNA is consistently present in brain tissue in several studies [62,101,105]. Also, pathological findings have been noted in the brain and behavior of infected hamsters. Bryce and colleagues reported that hamsters experienced damage to the olfactory epithelium as early as day 2 pi, resulting in exposure of axon bundles [102]. Anosmia has also been reported in several studies of infected hamsters associated with damage in the olfactory epithelium and resolution with viral clearance [101,106].

Extrapulmonary tissues such as the kidney, spleen, liver, pancreas, and gastrointestinal tract have been examined in the hamster model of acute COVID-19. Our assessment of the spleen demonstrated prominent immune responses increasing from day 8 to day 15 [70], correlating with typical splenic involvement during host responses to pathogens [107]. Depletion of white and red pulp with reduced number and size of follicles has also been reported [62,68]. Investigation of liver damage revealed systemic (although nonstatistical) increases in alkaline phosphatase (ALP), blood urea nitrogen (BUN) [69,107], and gamma-glutamyl transferase (GGT) [69]. Nausea and diarrhea have seldom been reported in hamsters infected with SARS-CoV-2, although viral RNA was present in the GI tract [70,108,109]. We found eosinophilic cytokines to be increased and eosinophils to be present in the large intestine [70]. We also found signs of kidney injury as CD14, IL-1β, and TNF-α were up-regulated early in the kidney coinciding with tubular injury seen in the histopathology and agreeing with other studies [58,70]. Most recently, the effects of SARS-CoV-2 on the skeletal system and bone metabolism have been investigated in the Syrian hamster model [110]. The authors found bone loss in the femur and tibia and demonstrated induction of osteoclastogenesis in hamsters during SARS-CoV-2 infection. Interestingly, they did not detect viral RNA or virus within the skeletal system but showed that proinflammatory cytokines (i.e., IL-1β and TNF-α) derived from the respiratory system were able to disrupt bone metabolism and promote osteoclastogenesis [110]. Lastly, despite viral RNA being present, pancreatitis has not been commonly observed in hamsters responding to SARS-CoV-2 infection [66,69]. Together, these studies suggest a possible multiorgan disease profile resulting from SARS-CoV-2 infection.

### Mouse models of SARS-CoV-2 infection

Due to similar biological features to humans, the highly characterized immune system, and rapid breeding and growth cycles, mice are extremely helpful in understanding human clinical disease [111]. However, mice are not susceptible to infection with the original emergent SARS-CoV-2 virus due to the poor binding between murine ACE2 and the viral spike protein [112]. Alternative approaches have therefore been developed to leverage mice for SARS-CoV-2 research.

One route was to adapt the SARS-CoV-2 virus to mice by sequential passaging in mouse lung tissue [113] or modifying the SARS-CoV-2 virus (mouse-adapted SARS-CoV-2) to be compatible with mouse ACE2 [112,114]. Both approaches have led to productive infection characterized by mild disease in wild-type mice. No weight loss was observed in BALB/c mice infected with mouse-adapted SARS-CoV-2, although virus replicated in the respiratory tract clearing by day 4 pi [114]. Increased disease severity can be achieved through additional mouse adaptations.

Alternatively, mice can be rendered susceptible to SARS-CoV-2 by genetically modifying the mice to express the human ACE2 (hACE2) gene. The hACE2 gene has been inserted into mice under the control of the endogenous mACE2 promoter [115], the cytokeratin-18 (*Krt18* (K18)) promoter for epithelial cell expression [78,80,82,116,117], or the *HFH4* (*FoxJ1*) promoter [118]. Mice expressing hACE2 by the endogenous mACE2 promoter express hACE2 in the lungs, heart, kidneys, and intestines [115]. After SARS-CoV-2 infection, these animals experience weight loss and lung inflammation; however, lung injury is limited (Table 1). No viral antigens or histopathological changes were observed in extrapulmonary tissues, although viral load was detected early in the intestine of some animals. K18-hACE2 C57BL/6J mice [strain 2B6.Cg-Tg(K18-ACE2)2Prlmn/J] [78,117,119] express hACE2 on epithelial cells and have up to 100-fold higher hACE2 mRNA expression compared to hACE2 knock-in (KI) mice in the lung and nasal turbinate [80]. K18-hACE2 mice exhibited a lethal respiratory disease; however, weight loss and mortality varied depending on the infectious dose [119]. *HFH4* (*FoxJ1*) promoter driven hACE2, on the other hand, results in overexpression of hACE2 in ciliated cells of the respiratory tract epithelium and central nervous system [114]. As expected, virus was detected in the lungs and brains of mice, with 40% mortality by day 5 pi. However, criticism for this model has been expressed due to the significant viral neuroinvasion. Additionally, hACE-2-KI (B6.129S2(Cg)-Ace2tm1(ACE2)Dwnt/J) mice have also been developed by substituting the *mACE2* gene with *hACE2* [83]. Winkler and colleagues demonstrated that hACE2 mRNA is expressed in the lung, nasal turbinate, kidney, duodenum, and olfactory bulb [80] but not in the colon, ileum, heart, spleen, or liver of the KI mice.

Although these genetically modified mice are susceptible to SARS-CoV-2 infection, the pathogenicity following SARS-CoV-2 varies from mild to lethal disease due to differences in hACE2 surface expression levels and tropisms may not fully replicate human expression [111]. It is also important to consider the development of encephalitis after SARS-CoV or SARS-CoV-2 intranasal infection in most of the mouse models, which does not accurately represent human disease [80,112]. Interestingly, aerosol inoculation of SARS-CoV-2 in K18-mice circumvents infection in the central nervous system suggesting a model for more appropriately recapitulating human features of COVID-19 [120].

Aside from germline manipulation of mice to express hACE2, another approach has been to transiently express hACE2 using adenovirus (AdV) or adeno-associated virus (AAV) expression systems [81,84]. In one study, BALB/c mice transduced with AdV-hACE2 and infected with SARS-CoV-2 had significant weight loss and high lung viral titers on day 4 pi, viral pneumonia, and low amounts of viral RNA in extrapulmonary tissues such as the heart, spleen, and brain [84]. Comparatively, SARS-CoV-2 replication in AAV-*hACE2* is lower [81], although both models can be helpful for SARS-CoV-2 research. It is important to note that cellular or tissue tropism of SARS-CoV-2 might change due to ectopically expressed hACE2 in these animals, leading to misinterpretation of the results.

Other potential SARS-CoV-2 mouse models include humanized mice or collaborative cross mice [121]. Human immune system (HIS)-humanized mice have components of the human immune system, express hACE2 and TMPRSS2 serine protease on lung epithelia, and are susceptible to SARS-CoV-2 [122]. On the other hand, MISTRG6 humanized mice in combination with AAV delivery of hACE2 to the lungs have been used to study post-acute COVID-19 [123]. The benefit of this model is that it reflects the human innate and adaptive responses to SARS-CoV-2. MISTRG6 humanized mice transiently expressing hACE2 displayed common features of post-acute sequelae such as lung pathology and sustained inflammatory responses up to 28 days pi [123]. In contrast to humanized mice, collaborative cross mice are a large panel of multiparental recombinant outbred mice designed to model genetic diversity and overcome the limitations of existing single-strain mouse model genetics. These mice are particularly useful for analysis of phenotypes caused by combinatorial allele effects, similar to humans although they have to be used in combination with mouse-adapted SARS-CoV-2 [121,124].

There is potential for mice to model the long-term consequences of SARS-CoV-2 infection. However, due to the varied viral dynamics and disease characteristics depending on the model, the power of such studies will be determined by the choice of model used.

### Ferret models of SARS-CoV-2 infection

The ferret (*Mustela putorius furo*) is well known to be susceptible to various human respiratory viruses such that they are often one of the first models to be utilized during an outbreak of a newly emerging respiratory virus. Ferrets are susceptible to respiratory syncytial virus (RSV), parainfluenzaviruses (PIV), SARS-CoV, and many others [125]. They have become the primary animal model for studying influenza viruses as well as evaluating influenza vaccines and therapeutics [125]. Ferrets have upper and lower respiratory tracts that proportionally resemble those of humans with a similar density of submucosal glands in the bronchial walls and a similar number of generations of terminal bronchioles rendering them the gold standard model for influenza studies [125]. At the physiological level, ferrets undergo similar immune maturation, respiratory development, and aging as humans, allowing age-related investigations of immunopathology [126–135]. It is generally considered that reagents are limited for immunological dissection in the ferret model. However, significant strides have been made in recent years to expand the ferret immunological toolbox with published studies focusing on the transcriptome, infectome, glycome, and B cell receptor repertoire, as well as T follicular helper cell identification [136–141].

Ferret ACE2 shares 82.6% sequence identity with humans [142] and ferrets were previously used to model SARS-CoV infection and vaccination [143–145]. Therefore, the ferret model was naturally applied early in the COVID-19 pandemic to determine the pathogenesis of SARS-CoV-2 infection and evaluate therapeutics. Although ferrets are susceptible to the SARS-CoV-2 virus [85,86,146], they display mild clinical disease characterized by minimal elevations in body temperature, minimal or no weight loss, little or no reduced activity, and mild acute bronchiolitis (Table 1) [85,87]. Live virus was restricted to the upper respiratory tract although viral RNA was detected in various tissues including the nasal turbinate, lung, trachea, intestine, kidney, as well as the urine and feces of infected animals [85]. Live virus peaked in ferrets on day 4 pi [85]. The prominent upper respiratory infection suggested the ferret to be useful for transmission studies and they have now demonstrated efficient aerosol and contact transmission of SARS-CoV-2 [85,87,146,147]. Our ferret study as well as others have found that nasal turbinate viral load was associated with rhinitis and nasal epithelium degeneration and necrosis [87,148]. Neutralizing antibodies against SARS-CoV-2 were apparent in some studies as early as day 8 pi correlating with virus clearance [85,87]. Our work indicated a delayed type I IFN responses in males that was exacerbated by age and was associated with prolonged viral replication in the upper respiratory tract. These findings complemented other aged ferret studies [149,150].

Taken together, ferrets do not display severe disease recapitulating the spectrum of COVID-19 disease severity seen in people. However, work in ferrets with SARS-CoV-2 has highlighted the contribution of host factors such as age and sex to disease sequelae as well as the transmission dynamics of SARS-CoV-2. Considering the knowledge gaps related to Long COVID, ferrets may not be the optimal model, although there may be some utility in dissecting the influence of host factors.

### COVID-19 nonhuman primate models

Nonhuman primates (NHPs) are an important preclinical model for understanding the pathogenesis of many respiratory viruses as well as other infectious and noninfectious diseases. NHPs such as rhesus macaques (*Macaca mulatta*), cynomolgus macaques (*Macaca fascicularis*), and African green monkeys (*Cercopithecus aethiops*) have been used for SARS-CoV-2 studies (Table 1) since they are more closely related to humans phylogenetically and immunologically than other animal models [111].

Early studies showed that rhesus macaques and cynomolgus macaques were susceptible to SARS-CoV-2 and were therefore utilized for vaccine and therapeutic studies. Both models had evidence of SARS-CoV-2 viral replication in the upper and lower respiratory tract by days 4 to 5, as demonstrated by the presence of sub-genomic viral RNA [88]. Furthermore, both macaque species exhibited mild to moderate disease with high neutralizing antibody titers by day 11 [88,89]. After infection, cynomolgus macaques had viral shedding from the nose and throat even in the absence of symptoms in some animals, similar to asymptomatic humans [88]. Infected cynomolgus macaques also displayed similar lung pathology to patients with mild/moderate COVID-19 characterized by alveolar necrosis and type II pneumocyte hyperplasia (Table 1) [88]. Older rhesus and cynomolgus macaques have also been used to dissect the impact of age on disease severity. More severe disease manifestations, such as viral pneumonia, have been detected in older animals compared to younger macaques [89,151]. In general, replication of SARS-CoV-2 in the respiratory tract, disease severity, and immune responses are similar among rhesus and cynomolgus macaques suggesting equal utility of the models [88]. African green monkeys infected with SARS-CoV-2 also display similarities to human disease, such as robust viral replication in the lungs and corresponding inflammatory lesions [93]. Live virus was highest in the lungs of African green monkeys from days 3 to 7 [93]. Current evidence indicates that African green monkeys may mount a more robust inflammatory response than macaques, differentiating this model from the macaque models [152].

In addition to clinical disease modeling, NHP models are useful for dissecting host response mechanisms associated with viral disease and immune protection. For example, African green monkeys infected with SARS-CoV-2 have similar immune profiles as compared to humans illustrated by increased IL-6 pi [93]. Similarly, increases in IL-6 are observed in the acute phase of macaque species. As well IFN-γ and chemokines (MIP-1α and MIP-1β) [90,91] are typically observed during SARS-CoV-2 infection [22]. Rhesus macaques exhibit differential airway responses depending on age, with up-regulated interferon-stimulated genes (ISGs) in the trachea of infants but not adults at day 14 pi [153]. Also in this study, adult rhesus macaques had higher serum IL-6 levels and transcriptomic profiling of the respiratory tract showed a profibrotic signature, possibly explaining why infants or younger individuals do not exhibit the same disease as adults [153]. With respect to immune pathogenesis, Böszörményi and colleagues analyzed immune mediators in rhesus and cynomolgus macaques during the post-acute phase of SARS-CoV-2 infection and found elevated IL-6 levels to be sustained in the serum at 5 to 6 weeks pi in some animals that corresponded to lung pathology [154]. A longer analysis showed that both adult African green monkeys and rhesus macaques with mild and moderate disease had inflammatory cells and lymphocytes in the lungs even at 4 weeks pi [155]. The sustained immune cell populations and inflammatory damage in the lungs of African green monkeys and rhesus macaques provide possible reasons for the prolonged pulmonary symptoms and delayed recovery of some COVID-19 patients.

## Potential for animal models in post-acute sequelae of COVID-19 studies

**Fig 2:**
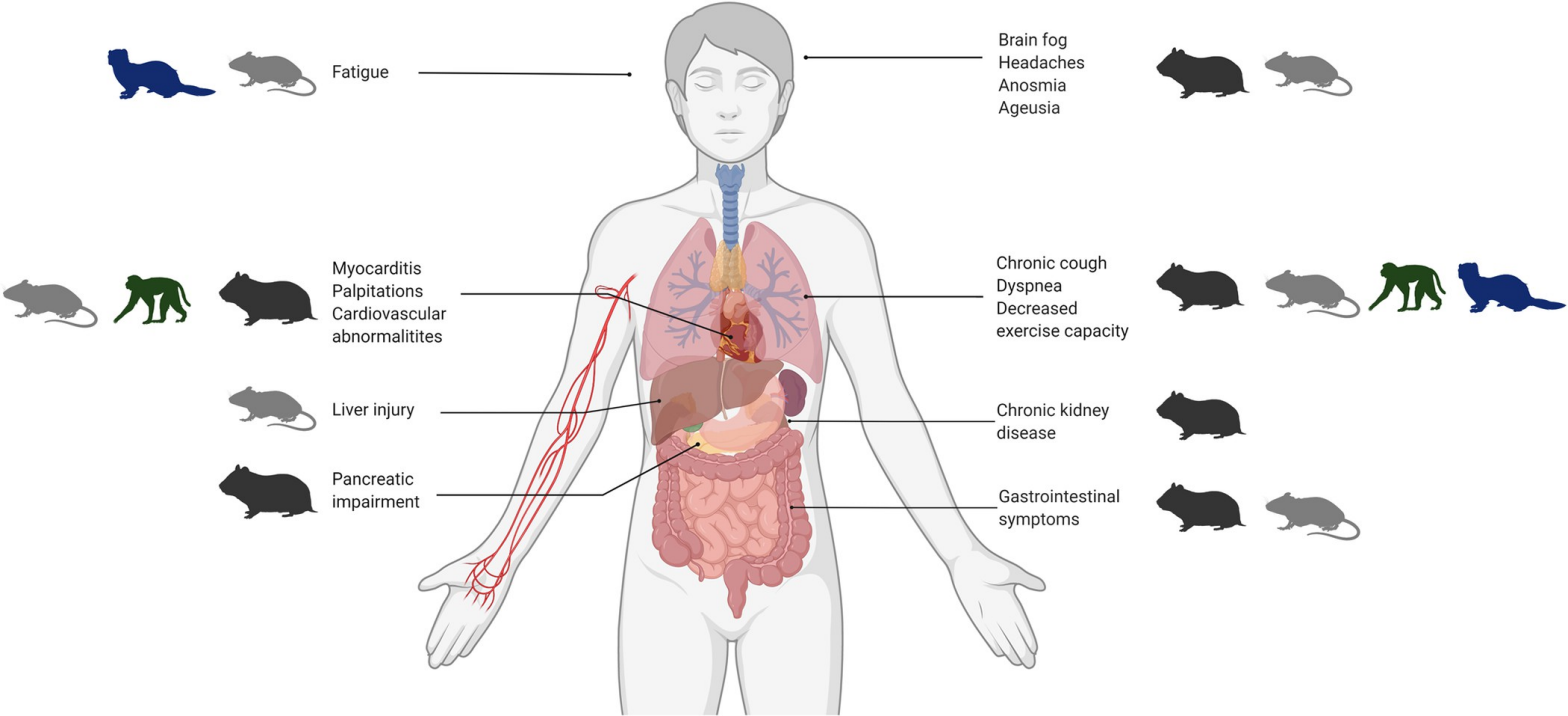
Potential animal models of post-acute sequelae of COVID-19 (PASC) manifestations. The 4 main groups of animal models (hamsters, mice, ferrets, and NHPs) are outlined with corresponding human manifestations of PASC. COVID-19, Coronavirus Disease 2019; NHP, nonhuman primate; PASC, Post-Acute Sequelae of COVID-19.

PASC is a multiorgan disease that affects the brain, lungs, heart, kidney, pancreas, spleen, liver, gastrointestinal tract, and blood vessels [33]. Several knowledge gaps exist regarding the cellular and molecular mechanisms leading to PASC [5]. In the sections above, we reviewed the clinical outcomes, pathology, and immune responses in several essential animal models following acute SARS-CoV-2 infection. To gain a view regarding how these models may be applied to Long COVID and PASC, we dissect these models by the most notable clinical parameters associated with Long COVID: prolonged respiratory disease; multiorgan pathogenesis and inflammation; cardiac and endothelial disease; neurological sequelae; and host risk factors of PASC. In reviewing how SARS-CoV-2 infection affects various tissues in preclinical model systems compared to human disease outcomes, we can assess the potential use of specific animal models for investigating aspects of PASC (Fig 2).

### Prolonged respiratory disease associated with clinical symptoms such as dyspnea and cough

The respiratory system is the first system affected by SARS-CoV-2 and is also significantly affected in PASC patients (Fig 1). The acute phase of SARS-CoV-2 infection in hamsters is comparable to humans in the context of respiratory disease. Moreover, hamsters have remaining lung damage after the acute phase that is applicable to the respiratory involvement in PASC [69,70,73]. Although more work is needed to examine the lungs of hamsters infected with SARS-CoV-2 at later time points, immune responses are unresolved after the virus is cleared which could indicate PASC-like lung characteristics [63,70,96]. Key inflammatory markers such as CXCL10 remain increased in hamsters on day 15 pi [96]. Since raised CXCL10 levels are linked to common symptoms of PASC, such as dyspnea and cough, hamsters may be an important option for the study of respiratory disease etiology associated with PASC [33–35]. NHPs such as macaques and African green monkeys also exhibit similar lung pathology to humans [90] and have been shown to have sustained immune cell infiltration in the lungs for up to 4 weeks pi [155]. Therefore, macaques and African green monkeys may be an effective model for understanding alveolar damage and fibrotic changes likely underlying dyspnea and chronic cough. Studies in rhesus and cynomolgus macaques identified pneumonia and lesions present in the lungs in the post-acute phase after viral RNA was no longer detectable in nasal and tracheal swabs [154]. Moreover, the authors of this study detected sub-genomic RNA, an indicator of viral replication, in the lungs during the post-acute phase and speculate that the detection of sub-genomic RNA after assumed resolution of infection indicates that pathogenesis is still occurring in macaques, presenting a possible model for investigating long-term consequences of SARS-CoV-2 infection in humans [154] (Fig 2). Conversely, ferrets developed only mild upper respiratory SARS-CoV-2 infection [112,146] instead of lower respiratory infection and damage as described in many humans. From these studies, ferrets may not be useful for investigating the underlying respiratory complications of PASC, such as dyspnea, decreased exercise capacity, and chronic cough. Due to the limitations discussed, it is unclear if mice will be a beneficial respiratory disease model. However, recent work in a humanized mouse model with human immune cells (MISTRG6) and hACE-2 expressing lungs was used to evaluate local and systemic pathology and immune responses 28 days post-SARS-CoV-2 infection [123]. The study found sustained viral RNA, weight loss without recovery, and lung fibrosis in the post-acute phase. The mice also had an aberrant inflammatory macrophage response and persistent ISG signatures suggesting the potential of this model for investigation of long-term respiratory pathogenesis; however, the transient expression of the ACE2 receptor may limit the usefulness.

### Multiorgan and systemic pathology, viral burden, and inflammation

Although initially a respiratory disease, the effects of SARS-CoV-2 infection affect several extrapulmonary organ systems, highlighting the need for a preclinical model to recapitulate the long-term multiorgan disease of COVID-19. The humanized mouse model (MISTRG6) and hACE-2 expressing lungs failed to recapitulate the multiorgan characteristics of PASC, suggesting its use in multiorgan disease may not be achievable [123]. Simpler models such as K18-hACE2 may be more useful for investigating chronic multiorgan complications since the K18 promoter allows for the expression of hACE2 in airway epithelial cells as well as epithelia of other extrapulmonary organs [117]; however, the infectious dose of SARS-CoV-2 should be considered to avoid lethal encephalitis and outcome [78,79,116]. K18-hACE2 mice exhibit lethargy during acute infection [116] and could be followed for reduced activity representing fatigue in the post-acute phase (Table 1, Fig 2). Additionally, in ferrets, viral RNA has been detected outside the respiratory tract in organs such as the gastrointestinal tissues, kidney, and brain, which may be relevant to the study of the multiorgan state of PASC, although the minimal lung infection should be considered [85,156]. The Syrian hamster has been used to study pathology across several organ systems, including less common symptoms of PASC such as pancreatitis, nausea, diarrhea, kidney injury, and splenic complications [68, 70]. Interestingly, Syrian hamsters also have progressive bone loss into the post-acute phase with no restoration of bone density by 60 dpi [110]. These studies suggest that the hamster model could be appropriate for evaluating extrapulmonary pathology associated with Long COVID.

Most of the SARS-CoV-2 pathogenesis modeled in NHPs has focused on the respiratory tract and systemic responses in the blood. However, the work done by Böszörményi and colleagues describes the replication and pathology of SARS-CoV-2 in respiratory and extra-respiratory tissues during the post-acute phase in both cynomolgus and rhesus macaques up to 6 weeks pi [154]. In addition to the respiratory tract, the authors detected viral RNA in the heart, lymph nodes, salivary gland, and conjunctiva during the post-acute phase. Another study reported similar detection of viral RNA in lymph nodes 21 days pi and in the nasal tissues for up to 27 days pi in some macaques [91]. Therefore, macaques may be a suitable model for investigating long-term immunopathology within lymph nodes and conjunctiva, in addition to the lungs. Furthermore, NHP models have inflammatory cytokines such as IL-6, which remain elevated into the post-acute phase, parallel to high systemic IL-6 levels in humans associated with severe COVID-19 [22,154]. Overall, macaques model the typical mild to moderate disease seen in COVID-19 patients [90] and display similar systemic immune profiles that may be useful in understanding the contribution of immune-mediated damage in post-acute sequelae if longer time points were investigated.

### Cardiac and endothelial disease

Symptoms of PASC commonly include heart palpitations, chest pains, myocarditis, and can less frequently include cardiovascular abnormalities [33–35]. These heart-related dysfunctions could be studied in the hamster model, as hamsters have exhibited cardiovascular damage in relation to SARS-CoV-2 infection [62,70]. While no histological changes were observed in the hearts of hamsters at 31 days pi in one study [77], another study has proposed hamsters as an effective model of CVCs based on their characterization of cardiovascular pathology up to 14 days pi [100]. They detected ventricular wall thickening, increased ventricular mass/body mass ratio, and interstitial coronary fibrosis in the late stages of acute infection (days 7 to 14). Infected hamsters also had increased serum cardiac troponin I, cholesterol, low-density lipoprotein, and a lipid profile similar to that of humans with CVCs [100]. NHP and mouse models may also be useful for investigating cardiac disease. Viral RNA has been detected in the hearts of macaques during the post-acute phase at 5 to 6 weeks after SARS-CoV-2 infection as well as in the hearts of hACE2 BALB/c mice [84,154]. Determining a mechanism of cardiovascular abnormalities will be essential for identifying who is susceptible to PASC, as well as how to treat it. At this point, the hamster may be the most suitable model so far to study PASC-associated cardiac involvement.

### Neurological issues

Neurological and cognitive-related issues are also associated with PASC. These include olfactory dysfunction, headaches, cognitive disturbances, and mood disturbances such as depression and anxiety [33–35]. While these symptoms could be measured in animal models of PASC, limited data regarding neuroinfectivity and neurological manifestations have been reported thus far. Behavioral studies using animal models may provide critical insights regarding the observable phenotypes of COVID-19, but choosing an appropriate model is challenging, particularly when the realities of working in containment level 3 are considered. While NHPs exhibit many remarkably similar behavioral phenotypes to humans, they are only accessible to a limited number of research groups, expensive to house, and have a long lifespan. In contrast, rodents are relatively easy to house in large numbers and mature quickly; thus they may be more feasible to study the long-term effects of SARS-CoV-2 on the brain and related symptomology. Although species-typical behavior is less characterized in hamsters than in mice, either species could be used in tasks to characterize olfactory disturbances, cognitive dysfunction, and mood-related symptomology [77,157]. However, care must be taken to select tasks that require minimal training and equipment given the restriction of working in containment level 3. Recently, our group has worked on optimizing spontaneous recognition memory tasks for use in hamsters using odors, objects, and social stimuli. Preliminary results suggest that untreated hamsters can discriminate between novel and familiar odor cues and social stimuli, setting the stage to explore the long-term effects of SARS-CoV-2 infection on this type of cognition [158].

Reports regarding acute olfactory dysfunctions, particularly in the Syrian hamster model, have been described in response to SARS-CoV-2 [101]; although, there is debate regarding whether SARS-CoV-2 mechanistically induces anosmia in humans via central or peripheral nervous system effects. Currently available data from hamster studies suggest that SARS-CoV-2 can cause massive olfactory epithelial damage but reports on whether olfactory sensory neurons themselves become infected are contradictory [102,159]. Interleukin-2 receptor subunit gamma (IL2RG) KO hamsters appear susceptible to SARS-CoV-2 neuroinvasion [65], but data from WT hamsters have been inconclusive [102,159]. A recent preprint explored the long-term (1 month) consequences of infection with SARS-COV-2 on the olfactory bulb and related behavior compared to Influenza A virus (A/California/04/2009) in age-matched and sex-matched Syrian hamsters [77]. Long-term assessment of the olfactory bulb in both groups of virally infected hamsters revealed that by 31 days pi, while animals recovered from influenza virus infection had returned to baseline, animals recovered from SARS-CoV-2 infection still had a robust type I interferon response and microglial activation despite lack of infectious virus or traces of viral RNA in any tissues, including the olfactory bulb itself. Olfactory-related behavior was examined in a food-finding test where the hamsters had to forage for food hidden in bedding material. Deficits in the SARS-CoV-2-infected hamsters were observed during the acute phase 3 days pi, but not 15 or 21 days pi [77]. In the same study, a marble burying test, typically used as a proxy for repetitive or compulsive-like behavior, conducted at 26 days pi revealed significant differences between SARS-CoV-2-infected hamsters and controls. These findings show that while basic olfactory function may return to normal in hamsters relatively soon after SARS-Cov-2 infection, more complicated behaviors may be disrupted for at least a few weeks. Additional data regarding the domains of cognition and related behavioral disturbances is urgently needed from the hamster model, as well as cross-species validation with mouse models.

Limited data regarding the neural circuits affected in hamsters following SARS-CoV-2 infection has been published. In the study discussed above, influenza virus infection and SARS-CoV-2 infection both led to an overall increase in the type I interferon response throughout the nervous system. However, region-specific changes were noted in the acute phase of infection with both viruses as well which should be considered in regard to long-term effects [77]. The striatum had notable alterations to metabolic function in the acute response to both viruses, whereas the thalamus had a virus-specific response. SARS-CoV-2 infection led to a decrease in thalamus activity, whereas influenza virus infection led to an increase in thalamus activity (3 days pi) [77]. As decreased function with the thalamus has been previously associated with cognitive deficits [160–162], Frere and colleagues postulated that this dampening of activity could be a cause of neurologic issues seen in cases of PASC [77]. Chronic inflammation in the olfactory bulb could affect a multitude of cognitive processes, including sensory [163,164] and emotional issues, perhaps inducing PASC-associated symptoms of loss of taste and smell [165,166], as well as depression [167,168]. Overall, this study by Frere and colleagues has made some important connections between PASC and the hamster model and offered hypotheses as to the biological causes of common symptoms. The hamster model will be essential in further exploring these hypotheses extending past the acute phase, as well as assessing treatment for the PASC-associated symptoms.

### Host factors influencing PASC and PASC symptoms

Clinical data has indicated that host factors influence the development of Long COVID. Therefore, it will be important that preclinical models are able to explore the influence of host factors such as sex, age, and comorbidities on disease.

As ferrets are outbred, have longer life spans, display human-like features of aging, and can be manipulated to manifest human chronic disease, they have long been used to dissect the influence of host factors on viral disease [127–129,136,169–171]. For COVID-19, ferrets are sensitive to several factors relevant to PASC in humans, including sex and age [149,150]. For example, the ferret model could be utilized to understand why females are reported to be more likely to experience certain Long COVID symptoms at 4 to 8 weeks pi [47]. Ferrets also become lethargic during acute infection [150], which may persist into chronic post-viral fatigue and disproportionately affect ferrets by sex. These aspects can be followed specifically by monitoring for reduced activity months after infection as we and others have conducted long-term ferret studies [172,173].

NHP models recapitulate COVID-19 with respect to age. Since young macaques typically exhibit mild disease [88,91,151], this model may be useful when trying to understand the long-term consequences of a typical mild or asymptomatic case in younger individuals who may go on to develop Long COVID complications or continuing sequelae even if the sequelae is mild. For example, a recent study has investigated age-related pathogenesis in macaques and found differences in respiratory tract responses to SARS-CoV-2 infection, with adult macaques exhibiting profibrotic gene signatures and lower expression levels of ISGs compared to young macaques [153]. Longer time points should be investigated to understand how the immunopathogenic differences in the acute phase resolve or continue. African green monkeys infected with SARS-CoV-2 also have disease severity that correlates with increased age [174]. These models may be leveraged to understand the role of age in Long COVID development; however, the use of NHPs should be considered with respect to their high cost, maintenance, low animal numbers, and facilities needed for the long-term observations.

## Implications for potential PASC therapeutics

There are a growing number of therapeutics in clinical trials for Long COVID, with over 400 registered on NIH.gov [175]. Most trials focus on treatments for pulmonary manifestations, with others in development for extra-respiratory complications such as neurological or CVCs [175]. However, our lack of understanding regarding the multiorgan pathogenic mechanisms of PASC makes the development of effective therapeutics challenging. Therefore, relevant PASC animal models will be crucial in developing novel therapeutics or repurposing available therapeutics to address the wide range of long-term symptoms. Some examples of therapeutics currently being tested in Long COVID patients include repurposed therapeutics for autoimmune diseases, anti-inflammatory treatments, and even dietary supplements.

A Phase II study of the ribonuclease RSLV-132 is underway in Long COVID patients (NCT04944121). RSLV-132 is an RNase-Fc fusion protein designed to treat autoimmune diseases such as lupus and Sjögren’s syndrome [176,177]. The drug digests extracellular RNA to prevent activation of the immune system via toll-like receptors and the interferon pathway [176]. A study in macaques detected sub-genomic viral RNA persisting in the lungs; therefore, this may be an interesting model to test RSLV-132’s efficacy and ability to prevent post-acute pathology [154]. Ferrets may also be used as viral RNA was detected throughout the body [149]. Sodium pyruvate nasal spray is another therapeutic currently in clinical trials for the treatment of Long COVID (NCT04871815). Sodium pyruvate impairs inflammatory cytokine production (IL-6, IL-1β, and TNF-α) in macrophages during Influenza A virus infection [178] and has been tested in clinical trials for acute COVID-19 and now Long COVID [179]. Results from a study of 22 Long COVID patients suggested improvements in headaches, coughing or sneezing, and breathing difficulties [179]. Unfortunately, this study was not randomized and contained no placebo controls. Further work is needed to assess sodium pyruvate in larger cohorts of people or preclinical models with appropriate controls. A potentially effective model to test sodium pyruvate would be Syrian hamsters due to their similarity in upper and lower respiratory tract infection with SARS-CoV-2 and similar multiorgan disease to that of humans [70]. As a nasal spray, it would be interesting to see the effect of sodium pyruvate on unresolved immune responses and damage in the lungs of hamsters after viral clearance [70,77]. Due to the presence of neurologic symptoms such as headaches, depression, and “brain fog,” a recent study is evaluating the efficacy of Niagen, a commercially available dietary supplement also known as nicotinamide riboside (NR) (NCT04809974). NR is a precursor for nicotinamide adenine dinucleotide (NAD+), which is an essential coenzyme used in various metabolic pathways and is a focus for the treatment of various cardiovascular, neurological, and metabolic disorders [180]. This study will assess whether NR can improve neuropsychological complications of Long COVID by measuring cognitive function, depression, anxiety, and physical symptoms. Hamsters have emerged as a potential model for neurological complications in the post-acute phase and could be used to test neurological therapeutics such as NR [77]. Animal models will be integral in assessing the hypothesized pathogenic role of persistent viral RNA, virus-induced autoimmunity, and prolonged inflammatory responses in PASC and, therefore, could also be useful for testing repurposed drugs such as RSLV-132, sodium pyruvate, and NR for treatment of various multiorgan complications. As we learn more about the underlying mechanisms of sequelae through animal models and human studies, further therapeutic targets and patient supports can be identified and improved for large-scale patient use.

## Conclusion

COVID-19 has significantly affected people’s health and well-being, possibly having a greater impact on community health than any other modern-day disease. As we strive to end the COVID-19 pandemic, it is essential to recognize that the continuation of COVID-19 in the form of Long COVID or PASC may hinder the goal of returning to pre-pandemic life long after the virus has been cleared. We recognize that preclinical animal models are essential for identifying molecular mechanisms driving disease as well as evaluating and discovering therapeutics for the disease. Long COVID is a multifaceted disease with many symptoms, which we analyzed across several animal models for extrapolation to human disease. Preclinical models are essential for moving potential pharmacological therapeutics through to clinical trials and also to support regulatory approval. In this review, we created a foundation for the use of preclinical models in Long COVID research to understand the disease etiology and to be applied to therapeutic discovery.
